# P-532. The burden of HMPV-associated hospitalization among children aged <18 years, 2016-2024

**DOI:** 10.1093/ofid/ofaf695.747

**Published:** 2026-01-11

**Authors:** Leah Goldstein, Anna Wang-Erickson, Ayzsa Tannis, Peter G Szilagyi, Geoffrey A Weinberg, Mary A Staat, Janet A Englund, Eileen J Klein, Julie A Boom, Jennifer E Schuster, Natasha B Halasa, Leila C Sahni, Laura S Stewart, Rangaraj Selvarangan, Marian G Michaels, Daniel C Payne, Fatimah S Dawood, Heidi L Moline, John Williams

**Affiliations:** Centers for Disease Control and Prevention, Atlanta, Georgia; University of Pittsburgh, Pittsburgh, PA; Centers for Disease Control and Prevention, Atlanta, Georgia; UCLA, Los Angeles, California; University of Rochester Sch Med & Dent, Rochester, New York; Cincinnati Children's Hospital Medical Center, Park Hills, Kentucky; Seattle Children’s Hospital/Univ. Washington, Seattle, Washington; Seattle Children's Hospital and University of Washington School of Medicine, Seatte, Washington; Baylor College of Medicine, Houston, Texas; Children's Mercy Kansas City, Kansas City, MO; Vanderbilt University Medical Center, Nashville, TN; Baylor College of Medicine and Texas Children's Hospital, Houston, Texas; Vanderbilt University School of Medicine, Nashville, Tennessee; Children’s Mercy Hospital, Kansas City, Missouri; University of Pittsburgh/ CHP, Pittsburgh, Pennsylvania; Cincinnati Children's Hospital Medical Center, Park Hills, Kentucky; Centers for Disease Control and Prevention, Atlanta, Georgia; US-CDC, Atlanta, Georgia; University of Wisconsin, Madison, Wisconsin

## Abstract

**Background:**

Human metapneumovirus (HMPV) is a major cause of respiratory disease. Clinical testing underestimates burden as HMPV is not included on most point-of-care tests. We estimated HMPV-associated pediatric hospitalization rates before and after the emergence of COVID-19 using clinical test results and prospectively collected surveillance specimens.

**Figure 1.**
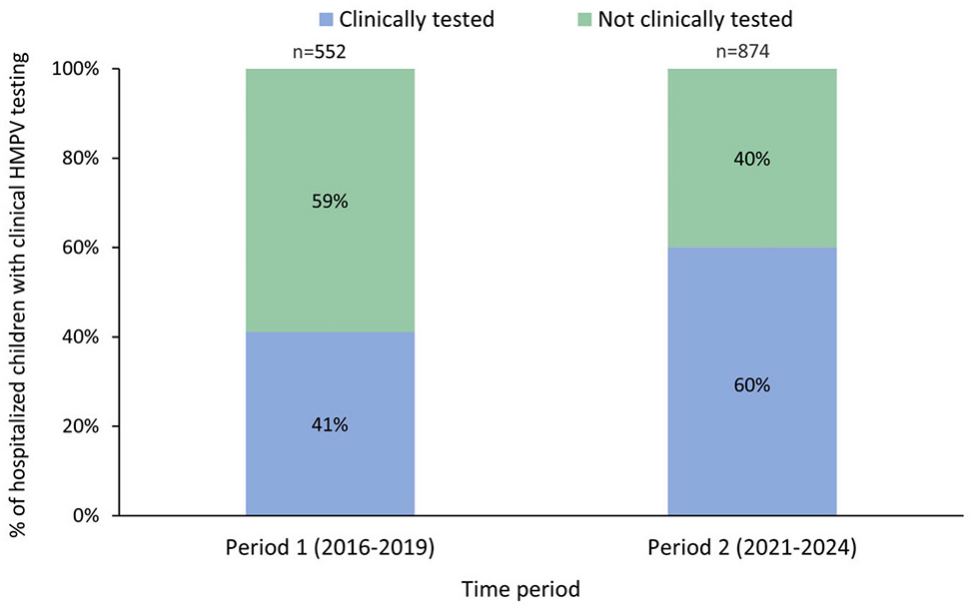
Proportion of hospitalized children with an HMPV-positive surveillance test who were also clinically tested, before and after the emergence of COVID-19

**Figure 2.**
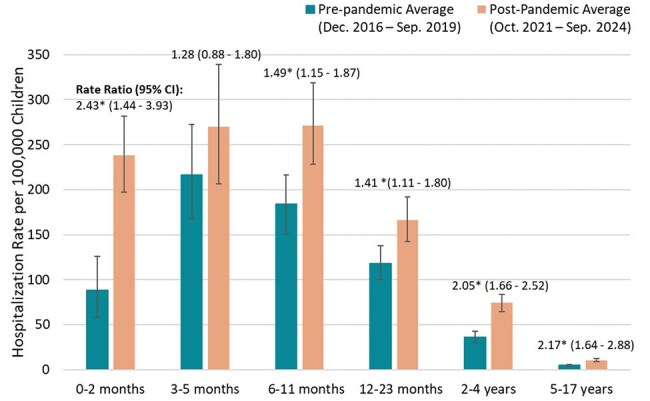
Estimated mean annual rate of HMPV-associated hospitalization and rate ratio comparing time periods, by age group * Indicates rate ratio differs at p-value <0.05 based on t test

**Methods:**

We enrolled children aged < 18 years hospitalized with acute respiratory illness (ARI) from December 1, 2016- September 30, 2019 (period 1) or October 1, 2021-September 30, 2024 (period 2) at 7 US medical centers in the New Vaccine Surveillance Network. Data sources included parent interviews, medical chart review, and clinical and surveillance HMPV test results for respiratory swabs. Mean annual rates from October-September and rate ratios were estimated per 100,000 children for each period using US census population denominators; 95% confidence intervals (CI) were estimated by bootstrap percentiles based on 1,000 bootstrap samples. Rates were adjusted for enrollment rates, < 7 surveillance days per week, and hospital market share. Rate ratios were calculated and compared using t tests and considered significant at P< 0.05.

**Results:**

599 children with HMPV identified by surveillance or clinical testing were enrolled during period 1, and 964 during period 2. During period 1, 41% of patients with a HMPV-positive surveillance test were clinically tested for HMPV, which increased to 60% during period 2 (Figure 1). Mean annual HMPV hospitalization rates were highest among children aged 3-5 months and 6-11 months in most years. Rates in all age groups increased significantly from period 1 to 2 except among children aged 3-5 months. The greatest change in HMPV rates was among 0–2-month-olds (RR 2.43, 95% CI 1.44-3.93, P < 0.001) (Figure 2).

**Conclusion:**

HMPV-associated hospitalization rates increased from before to after the emergence of COVID-19 in most age groups, with the highest rates among children aged 3-5 months and 6-11 months in both periods. A large fraction of hospitalized children with HMPV did not receive clinical testing, emphasizing the need for surveillance with systematic testing to estimate true HMPV disease burden.

**Disclosures:**

Geoffrey A. Weinberg, MD, Inhalon Biopharma: Advisor/Consultant|Merck & Co: Honoraria Mary A. Staat, MD, MPH, Centers for Disease Control and Prevention: Grant/Research Support|Cepheid: Grant/Research Support|Merck: Advisor/Consultant|Merck: Grant/Research Support|National Institutes of Health: Grant/Research Support|Up-To-Date: Royalties Janet A. Englund, MD, AstraZeneca: Board Member|AstraZeneca: Grant/Research Support|Cidarra: Member Data Safety Monitoring Board|GlaxoSmithKline: Advisor/Consultant|GlaxoSmithKline: Grant/Research Support|Meissa Vaccines: Advisor/Consultant|Merck: Advisor/Consultant|Merck: Grant/Research Support|Moderna: Advisor/Consultant|Moderna: Grant/Research Support|Pfizer: Advisor/Consultant|Pfizer: Grant/Research Support|Shionogi: Grant/Research Support Natasha B. Halasa, MD, CSL-Seqirus: Advisor/Consultant|Merck: Grant/Research Support Rangaraj Selvarangan, PhD, Altona: Grant/Research Support|Biomerieux: Advisor/Consultant|Biomerieux: Grant/Research Support|Biomerieux: Honoraria|Cepheid: Grant/Research Support|Hologic: Grant/Research Support|Hologic: Honoraria|Meridian: Grant/Research Support|Qiagen: Grant/Research Support Marian G. Michaels, MD, MPH, Merck: Grant/Research Support Daniel C. Payne, PhD, MSPH, Merck: Advisor/Consultant|Moderna: Advisor/Consultant

